# Analysis of the outcome of young age tongue squamous cell carcinoma

**DOI:** 10.1186/s40902-017-0139-8

**Published:** 2017-12-25

**Authors:** Jae-Ho Jeon, Min Gyun Kim, Joo Yong Park, Jong Ho Lee, Myung Jin Kim, Hoon Myoung, Sung Weon Choi

**Affiliations:** 1 Department of Oral and Maxillofacial Surgery, Seoul National University Dental Hospital, Seoul, Korea; 2 Oral Oncology Clinic, Research Institute and Hospital, National Cancer Center, 323 Ilsan-ro, Ilsandong-gu, Goyang-si, 10408 Gyeonggi-do Republic of Korea; 3 Dental Research Institute, Seoul National University, Seoul, Korea

**Keywords:** Tongue cancer, Young patients, Distant metastasis

## Abstract

**Background:**

The incidence of tongue squamous cell carcinoma (TSCC) in young patients has recently increased, and these TSCCs are believed to be etiologically distinct from those in older patients, who have longer exposure to risk factors such as tobacco and alcohol. The prognosis of TSCCs in young patients remains controversial.

**Methods:**

We retrospectively reviewed the records of 117 patients (2001–2011) who were diagnosed with squamous cell carcinoma of the oral tongue. Patients were divided into two age groups, older (ages over 40) and younger (ages 40 and younger). Data were compared between the two groups, and survival rates were analyzed.

**Results:**

The results show that there are significant differences in overall, disease-free, and distant metastasis-free survival rates between the two groups. Five-year overall survival rates were 70% in older patients and 42% in young patients (*p* = 0.033). Five-year disease-free survival rates were 73% in older patients and 40% in young patients (*p* = 0.011), and 5-year distant metastasis-free survival rates were 97% in older patients and 62% in young patients (*p* = 0.033).

Multivariate analysis revealed that histologic grade was the only independent risk factor for overall survival in both groups of patients (*p* = 0.002, HR = 2.287). The analysis also demonstrated that age was the critical risk factor for distant metastasis (*p* = 0.046, HR = 9.687).

**Conclusion:**

In this study, young (ages 40 and younger) patients with squamous cell carcinoma of the oral tongue had a higher rate of distant metastasis and a worse prognosis. Accordingly, we propose the necessity of an extensive therapeutic regimen that should be used in all young patients with TSCC.

## Background

Tongue squamous cell carcinoma (TSCC) comprises a small portion of all malignant cancer among patients. According to the previous data, about 45% of all oral cavity cancers were TSCC in a discrete period of study. TSCC normally affects males 60 to 80 years of age. Patients younger than 40 years with TSCC are considered young patients and are a small proportion of total TSCC patients. TSCC in young patients is not typically due to direct risks of exposure to smoking and drinking alcohol. TSCC in young patients is rare and is believed to be etiologically distinct from TSCC in older patients. However, the incidence of TSCC in young patients has recently increased [1–3].

The mobile tongue is the most common location of the head and neck cancer. It is controversial to state that the tongue cancer outcomes in young patients (40 or under 40 years) are better than the outcomes in older patients (over 40 years). Many studies in fact support the conclusion that young patients have worse outcomes than older TSCC patients [4–10]. Many authors have reported that more aggressive approaches are needed for TSCC patients less than 40 years of age in cases of recurrence or distant metastasis. Sarkaria and Harari [4] introduced the idea that prognosis in young patients was worse than prognosis in older patients. Byers [5] compared oral tongue cancer patients younger than 30 years of age with older patients. He proposed that the treatment of young patients with tongue cancer must be based on tumor factors and not on emotional factors. On the other hand, in a series of 27 patients with oral tongue and oral cavity cancer, McGregor et al. [8] found higher survival rates in young patients than in their adult counterparts.

The purpose of this study was to analyze the clinical characteristics and prognosis of young patients with TSCC in comparison to clinical characteristics and prognosis in a group of older patients in order to investigate whether onset age is an adverse factor for patients with TSCC.

## Methods

We retrospectively reviewed the records of 117 patients between May 2001 and August 2011 who were diagnosed with TSCC in the Department of Oral Oncology at the National Cancer Center in South Korea. Patients were divided into two age groups, older (ages over 40) and younger (ages 40 and younger). Study factors including patient sex; smoking history (Hx.); alcohol Hx.; cancer stages such as T stage, N stage, and TNM stage (American Joint Committee on Cancer, 6^th^ Edition, 2010); positive nodes; sites of recurrence; lymphovascular invasion; perineural invasion; perinodal extension; treatment; and histological grade were compared between the two groups.

### Statistical analysis

Statistical procedures included chi-square analyses and the Kaplan-Meier method, which contained overall survival, disease-free survival, and distant metastasis-free survival rates by age. Each survival curve was univariate analyzed by log-rank tests. Finally, Cox proportional hazards regression was used to determine risk factors.

## Results and discussion

From May 2001 to August 2011, 23 patients with ages of 40 years or younger(15 male and 8 female) and 94 patients with ages of over 40 years (51 male and 43 female) were treated underdiagnosis of TSCC at the National Cancer Center (Goyang, South Korea). The patients in both groups were Asian. The median age of the patients was 54 years, and the average age was 55.07 years (the range was 19 to 92 years) for the two groups. The average follow-up period was 33.56 months (the range was 2 to 124 months), and the median follow-up period was 20 months for the two groups. The last follow-up date was November 21, 2011. Clinical characteristics are presented in Table 1. Clinical characteristics in the two groups were similar except for histological grade (*p* = 0.021) and recurrence pattern of disease (*p* = 0.000). The two groups had no significant differences in the categories of sex; T stage; N stage; positive nodes; TNM stage; I, II/III, and IV stages; lymphovascular invasion; perineural invasion; and perinodal extension. Even though *p* values were greater than 0.05, there were differences of the proportion of TNM stage (*p* = 0.055) and perineural invasion (*p* = 0.082) between the two groups. Approximately 60.8% of the group of young patients presented with an advanced stage of the disease (stage III/IV), whereas 39.4% of the older patients presented with advanced-stage disease. In addition, about 14.9% of patients in the group of older patients presented with perineural invasion. On the other hand, 30.4% of patients in the group of young patients presented with perineural invasion.

**Table 1:** Clinical characteristics and statistical results for the two patient groups by age

		Age < 40 (*n* = 23) Number (%)	Age ≥ 40 (*n* = 94) Number (%)	All (*n* = 117) Number (%)
Sex *(p* = .342)	Male	15 (65.2)	51 (54.3)	66 (56.4)
	Female	8 (34.8)	43 (45.7)	51 (43.6)
Smoker *(p* = .460)	Yes	12 (52.2)	41 (43.6)	53 (45.3)
	No	11 (47.8)	53 (56.4)	64 (54.7)
Alcohol use *(p* = .786)	Yes	11 (47.8)	42 (44.7)	53 (45.3)
	No	12 (52.2)	52 (55.3)	64 (54.7)
T stage *(p* = .088)	T1	3 (13.0)	36 (38.3)	39 (33.3)
	T2	17 (73.9)	43 (45.7)	60 (51.3)
	T3	2 (8.7)	11 (11.7)	13 (11.1)
	T4	1 (4.3)	4 (4.3)	5 (4.3)
N stage *(p* = .154)	N0	11 (47.8)	64 (68.1)	75 (64.1)
	N1	3 (13.0)	13 (13.8)	16 (13.7)
	N2	9 (39.0)	17 (18.1)	26 (22.2)
	N3	0	0	0
Positive node *(p* = .177)	0	11 (47.8)	64 (68.1)	75 (64.1)
	1	3 (13.0)	13 (13.8)	16 (13.7)
	2	3 (13.0)	5 (5.3)	8 (6.8)
	≤ 3	6 (26.0)	12 (12.8)	18 (15.4)
TNM stage *(p* = .055)	I	2 (8.7)	33 (35.1)	35 (30.0)
	II	7 (30.4)	24 (25.5)	31 (26.5)
	II	4 (17.4)	16 (17.0)	20 (17.1)
	IV (a, b)	10 (43.4)	21 (22.3)	31 (26.5)
I, II, II, IV stage *(p* = .062)	I, II	9 (39.0)	57 (60.6)	66 (56.4)
	III, IV	14 (60.8)	37 (39.4)	51 (43.6)
Lymphovascular invasion (*p* = .760)	Yes	4 (17.4)	19 (20.2)	23 (19.7)
	No	19 (82.6)	75 (79.8)	94 (80.3)
Perineural invasion (*p* = 0.082)	Yes	7 (30.4)	14 (14.9)	21 (17.9)
	No	16 (69.5)	80 (85.1)	96 (82.1)
Perinodal extension (*p* = 0.632)	Yes	3 (13.0)	9 (9.6)	12 (10.3)
	No	20 (87.0)	85 (90.4)	105 (89.7)
Treatment *(p* = 0.247)	Surgery	8 (34.8)	47 (50.0)	55 (47.0)
	Surgery + radiotherapy	14 (60.8)	36 (38.3)	50 (42.6)
	Radiotherapy	1 (4.3)	10 (11.7)	11 (9.4)
Histological grade *(p* = 0.021)	Well	10 (43.5)	59 (62.8)	69 (59.0)
	Moderate	6 (26.0)	27 (28.7)	33 (28.2)
	Poor	6 (26.0)	5 (5.3)	11 (9.4)
	Unknown	1 (4.3)	3 (3.2)	4 (3.4)

A total of 37 (31.5%) patients developed recurrence. Recurrence patterns observed in young and older patients are presented in Table 2. Local recurrence was observed in 0 of 23 young patients in comparison to 2 of 94 patients in the older patient population. The regional recurrence rate was lower in young patients. One of 23 (4.3%) young patients showed regional recurrence in comparison to 9 of 94 (9.6%) patients in the older group. Six of 23 (26.0%) young patients had a locoregional failure in comparison to 11 of 94 (11.7%) older patients. Six of 23 (26.0%) young patients developed metastatic disease in contrast to 2 of 95 (2.1%) older patients with metastatic disease. The results represent higher locoregional recurrence and distant metastasis in young patients in comparison to older patients.

**Table 2:** Recurrence patterns in young and older patients

Site of recurrence*, p* = 0.000	Age < 40 (*n* = 23) Number (%)	Age ≥ 40 (*n* = 94) Number (%)	All (*n* = 117) Number (%)
Local recurrence	0	2 (2.1)	2 (1.7)
Regional recurrence	1 (4.3)	9 (9.6)	10 (8.5)
Locoregional recurrence	6 (26.0)	11 (11.7)	17 (14.5)
Distant metastasis	6 (26.0)	2 (2.1)	8 (6.8)

**Fig. 1:**
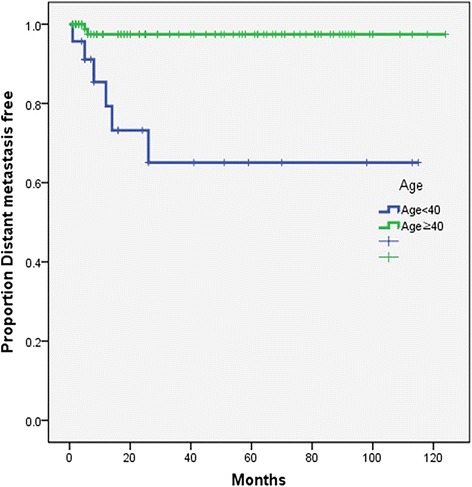
Kaplan-Meier overall survival curve (log-rank *p* = 0.033)

**Fig. 2:**
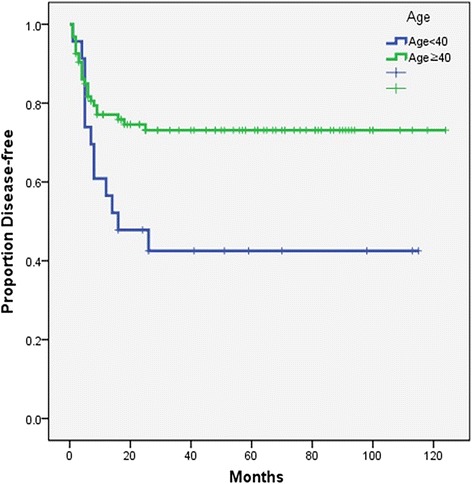
Kaplan-Meier disease-free survival curve (log-rank *p* = 0.011)

**Fig. 3:**
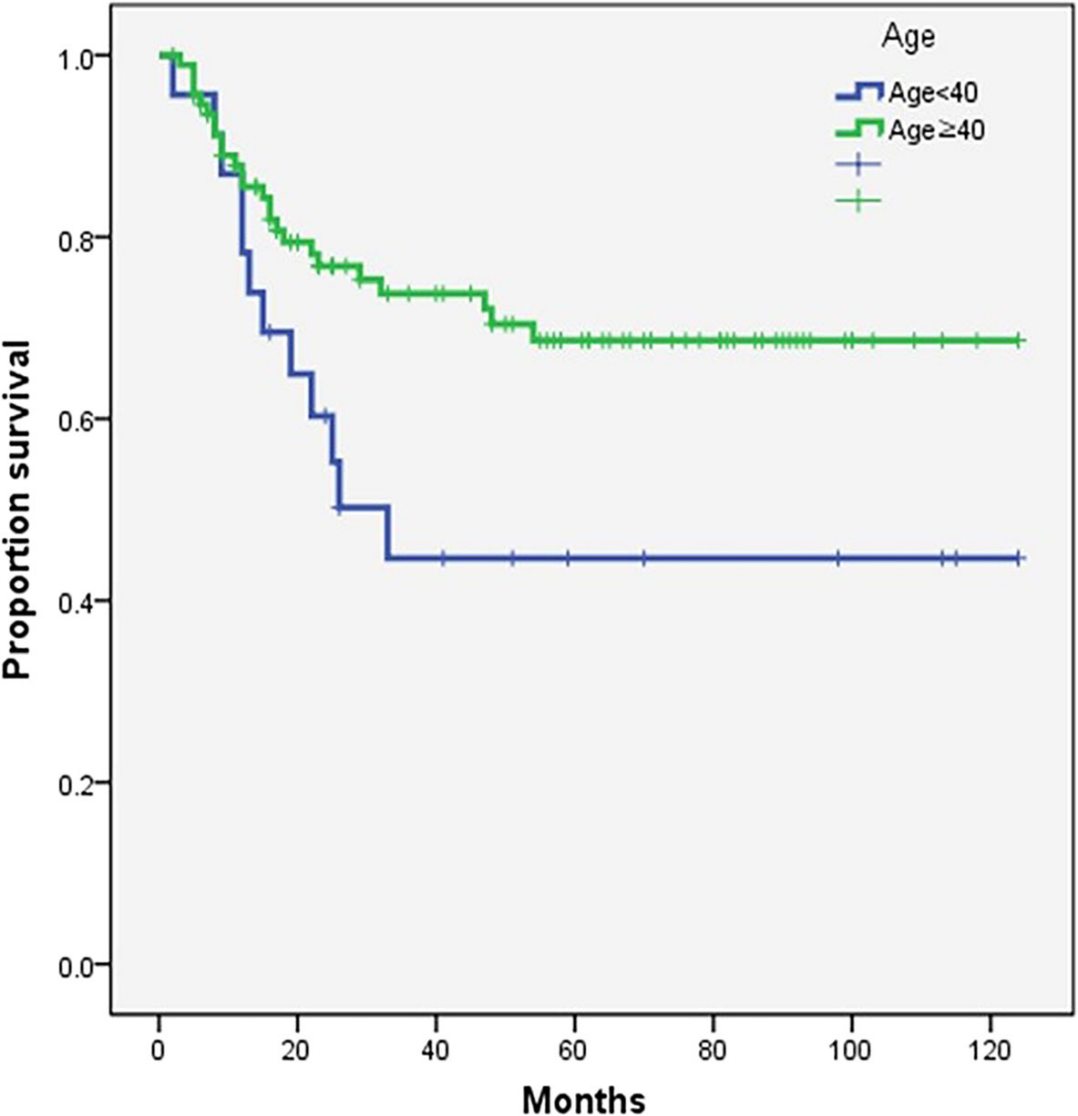
Kaplan-Meier distant metastasis-free survival curve (log-rank *p* = 0.033)

The results show that there are significant differences in overall, disease-free, and distant metastasis-free survival rates between the two groups. Five-year overall survival rates were 70% in older patients and 42% in young patients (*p* = 0.033) (Fig. 1). Five-year disease-free survival rates were 73% in older patients and 40% in young patients (*p* = 0.011) (Fig. 2), and 5-year distant metastasis-free survival rates were 97% in older patients and 62% in young patients (*p* = 0.033) (Fig. 3).

Multivariate analysis revealed that histologic grade was the only independent risk factor for overall survival rates in all patients (Table 3). In addition, the analysis showed that age was the critical risk factor for distant metastasis (Table 4).

**Table 3:** Multivariate analysis of risk factors for overall survival

Survival	*p* value	Hazard ratio	95% CI
Age	0.852	0.917	0.371–2.270
T stage	0.173	1.534	0.829–2.839
N stage	0.848	1.229	0.149–10.141
Node (*n*)	0.729	1.191	0.443–3.200
Lymphovascular invasion	0.479	0.718	0.287–1.798
Perinodal extension	0.678	1.255	0.429–3.667
Perineural invasion	0.712	1.167	0.515–2.646
I, II/III, IV stage	0.451	1.318	0.643–2.702
Grade	0.002	2.287	1.340–3.903
Smoker	0.674	0.831	0.352–1.966
Alcohol use	0.723	0.847	0.340–2.115

**Table 4:** Multivariate analysis of risk factors for distant metastasis

Distant metastasis	*p* value	Hazard ratio	95% CI
Age	0.046	9.687	1.042–90.081
T stage	0.196	2.279	0.654–7.942
N stage	0.420	13.276	0.025–7139.458
Node (*n*)	0.747	0.639	0.042–9.693
Lymphovascular invasion	0.379	2.525	0.321–19.851
Perinodal extension	0.274	3.490	0.372–32.739
Perineural invasion	0.629	1.628	0.226–11.719
I, II/III, IV stage	0.957	123.182	0.000–1.769
Grade	0.203	2.394	0.625–9.178
Smoker	0.686	0.608	0.055–6.774
Alcohol use	0.909	0.839	0.041–14.037

The work of Bachar et al. [11] reported that tumor depth and histological grade were worse in patients with exposure to smoking and alcohol risk factors. The authors’ analysis of young patients showed lower overall survival and disease-free survival in habitual non-smokers and alcohol drinkers than in habitual smokers and alcohol drinkers. The research suggests that an alternative pathogenesis of TSCC in young patients involves factors other than smoking and alcohol risk factors. In our research, a similar proportion of young and older patients had exposure to smoking and alcohol risk factors, but young patients tended to have a worse prognosis than older patients. Further studies are needed to investigate the molecular etiology and risk factors of tongue cancer in young patients.

Some controversy has existed as to whether young patients with squamous cell carcinoma of the oral tongue need more aggressive treatment than older patients. Many authors have reported that young patients with tongue squamous cell carcinoma show poor progress with worse survival rates than older patients and therefore need more aggressive treatment than older patients. A study from 1994 by Sarkaria and Harari [4] suggested that the outcomes (53% cause-specific survival) in young patients with tongue cancer were worse than the outcomes in older patients with tongue cancer. However, many authors have reported that young patients show no differences from older patients with TSCC in terms of outcomes. A study by Friedlander et al. [12] described no significant differences in survival rates between the two age groups. A recent report by Pitman et al. [13] supported similar outcomes between the two age groups with clinical data on 94 young patients in comparison to a control group of 150 older patients. The authors concluded that both groups had similar outcomes. In contrast, McGregor et al. [8] reported that young patients with squamous cell carcinoma of the oral tongue (SCCOT) showed better progress than older patients with SCCOT. Davidson et al. [14] analyzed two patient groups by age according to the Surveillance, Epidemiology, and End Results (SEER) tumor registry database with 749 patients. The authors reported that the risk of disease-specific death increased by 18% with every 10 years of age. Many studies have compared patient groups with the criteria of age (thereby dividing patients by age). However, it is difficult to be certain that age criteria reflect differences of age-specific tongue cancer.

Tongue cancer in young patients occurs without obvious risk factors. Many studies have statistical limitations with small sample sizes. Oral tongue cancer in young patients has shown a remarkable increase in comparison to other head and neck cancers in young patients. This study attempted to overcome these limitations. We identified 23 patients younger than 40 years of age and 94 patients over 40 years of age with SCC of the oral tongue. The patients were treated from 2000 to 2011. The two groups were compared in terms of sex, TNM stage, positive node, local and regional recurrence, and distant metastasis. A total of 37 of 117 (31.5%) patients experienced a recurrent event. The overall proportion of patients experiencing recurrence was lower than in other studies, with reported failure rates of 40%. Analysis of the two groups revealed that parity in sex, TMN stage, and positive nodes showed similar outcomes in both groups. However, distant metastasis was significantly different (in terms of statistical significance) between the two groups. Overall survival, disease-specific survival, and distant metastasis-free survival by age were significantly different between the two groups. Young patients had an increased risk of locoregional recurrence and distant metastasis. A recent study in 2006 by Chun-Ta Liao et al. [6] reported similar findings to the findings of our study. The authors reported that a group of young patients (≤ 40 years, *n* = 76) had a higher distant failure rate in comparison to a group of elderly patients (> 40 years, *n* = 220).

In the review by Turi et al. [15], while the cause of young age tumor is unclear, many studies agree that social factors such as smoking and alcohol consumption play a minimal role in the etiology. Some suggest that individual genetic factor and oncogenic genotype of HPV infection may play a more significant role.

In our research, the etiology of tongue cancer in young patients is exactly unknown, and this study is limited in its small sample size and the various inherent biases of a retrospective study.

## Conclusion

We found evidence of age-specific young cancer patients with a high risk of distant metastasis. Treatment for young patients with tongue cancer should be more aggressive than the treatment for older patients with tongue cancer. Although this study reveals the risk of distant metastasis in young patients with tongue cancer, clinical and pathological studies have been insufficient in terms of evidence for age-specific outcomes in patients with TSCC. Further studies are needed to investigate the etiology and risk factors of tongue cancer in young patients.

## Abbreviations

TSCC: Tongue squamous cell carcinoma
